# Contribution of intestinal triglyceride-rich lipoproteins to residual atherosclerotic cardiovascular disease risk in individuals with type 2 diabetes on statin therapy

**DOI:** 10.1007/s00125-023-06008-0

**Published:** 2023-09-29

**Authors:** Marja-Riitta Taskinen, Niina Matikainen, Elias Björnson, Sanni Söderlund, Jussi Inkeri, Antti Hakkarainen, Helka Parviainen, Carina Sihlbom, Annika Thorsell, Linda Andersson, Martin Adiels, Chris J. Packard, Jan Borén

**Affiliations:** 1 Research Programs Unit, Clinical and Molecular Medicine, University of Helsinki, Helsinki, Finland; 2 Endocrinology, Abdominal Center, Helsinki University Hospital, Helsinki, Finland; 3 Department of Molecular and Clinical Medicine, Institute of Medicine, University of Gothenburg, Gothenburg, Sweden; 4 HUS Medical Imaging Center, Radiology, Helsinki University Hospital, University of Helsinki, Helsinki, Finland; 5 Proteomic Core Facility at Sahlgrenska Academy, University of Gothenburg, Gothenburg, Sweden; 6 Institute of Cardiovascular and Medical Sciences, University of Glasgow, Glasgow, UK; 7 Wallenberg Laboratory, University of Gothenburg, Sahlgrenska University Hospital, Gothenburg, Sweden

**Keywords:** Apolipoprotein B-100, Apolipoprotein B-48, Chylomicrons, Liver, Metabolism, Postprandial, Stable isotope, VLDL

## Abstract

**Aims/hypothesis:**

This study explored the hypothesis that significant abnormalities in the metabolism of intestinally derived lipoproteins are present in individuals with type 2 diabetes on statin therapy. These abnormalities may contribute to residual CVD risk.

**Methods:**

To investigate the kinetics of ApoB-48- and ApoB-100-containing lipoproteins, we performed a secondary analysis of 11 overweight/obese individuals with type 2 diabetes who were treated with lifestyle counselling and on a stable dose of metformin who were from an earlier clinical study, and compared these with 11 control participants frequency-matched for age, BMI and sex. Participants in both groups were on a similar statin regimen during the study. Stable isotope tracers were used to determine the kinetics of the following in response to a standard fat-rich meal: (1) apolipoprotein (Apo)B-48 in chylomicrons and VLDL; (2) ApoB-100 in VLDL, intermediate-density lipoprotein (IDL) and LDL; and (3) triglyceride (TG) in VLDL.

**Results:**

The fasting lipid profile did not differ significantly between the two groups. Compared with control participants, in individuals with type 2 diabetes, chylomicron TG and ApoB-48 levels exhibited an approximately twofold higher response to the fat-rich meal, and a twofold higher increment was observed in ApoB-48 particles in the VLDL_1_ and VLDL_2_ density ranges (all *p *< 0.05). Again comparing control participants with individuals with type 2 diabetes, in the latter, total ApoB-48 production was 25% higher (556 ± 57 vs 446 ± 57 mg/day; *p *< 0.001), conversion (fractional transfer rate) of chylomicrons to VLDL was around 40% lower (35 ± 25 vs 82 ± 58 pools/day; *p*=0.034) and direct clearance of chylomicrons was 5.6-fold higher (5.6 ± 2.2 vs 1.0 ± 1.8 pools/day; *p *< 0.001). During the postprandial period, ApoB-48 particles accounted for a higher proportion of total VLDL in individuals with type 2 diabetes (44%) compared with control participants (25%), and these ApoB-48 VLDL particles exhibited a fivefold longer residence time in the circulation (*p *< 0.01). No between-group differences were seen in the kinetics of ApoB-100 and TG in VLDL, or in LDL ApoB-100 production, pool size and clearance rate. As compared with control participants, the IDL ApoB-100 pool in individuals with type 2 diabetes was higher due to increased conversion from VLDL_2_.

**Conclusions/interpretation:**

Abnormalities in the metabolism of intestinally derived ApoB-48-containing lipoproteins in individuals with type 2 diabetes on statins may help to explain the residual risk of CVD and may be suitable targets for interventions.

**Trial registration:**

ClinicalTrials.gov NCT02948777.

**Graphical Abstract:**

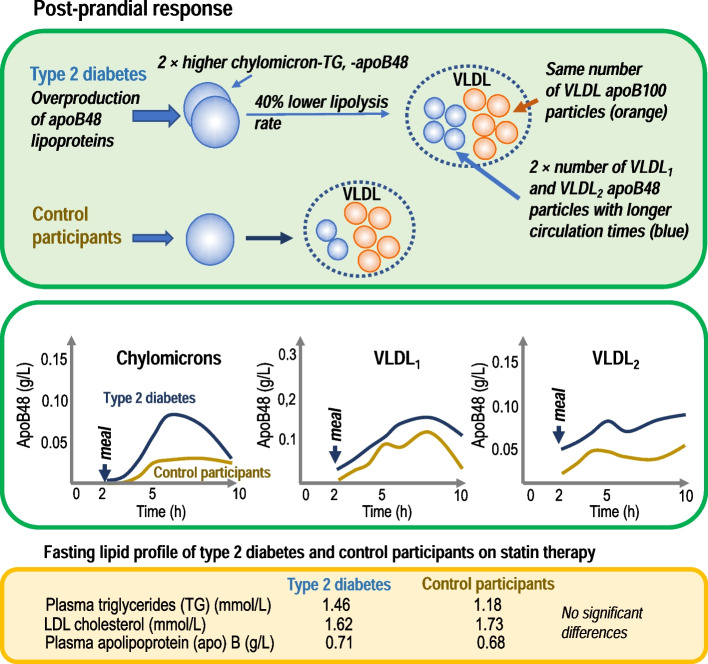

**Supplementary Information:**

The online version of this article (10.1007/s00125-023-06008-0) contains peer-reviewed but unedited supplementary material.


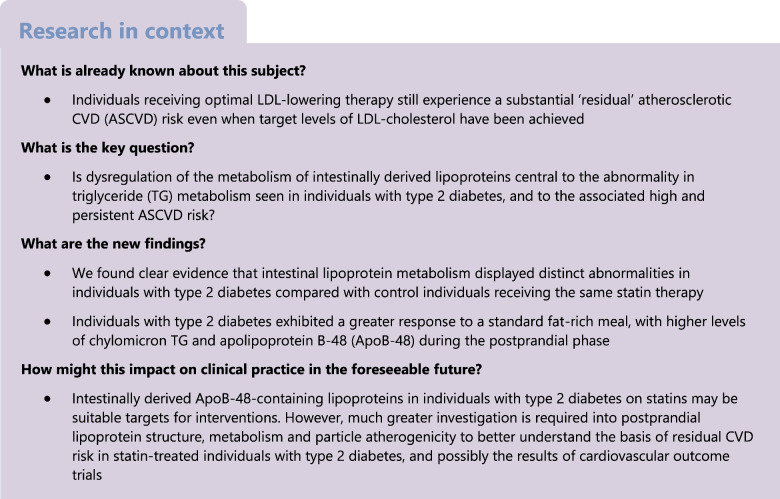


## Introduction

Clinical trials conducted over the last 25 years have established statins, and more recently combination therapy with statins, as the cornerstone of atherosclerotic CVD (ASCVD) prevention in a wide range of patient types [1]. A primary focus has been to lower LDL intensively to meet ever more aggressive goals as set out in international guidelines [2–4]. The same clinical trials, however, demonstrate that when individuals are receiving what is considered optimal LDL-lowering therapy, they still experience a substantial ‘residual’ ASCVD risk [5], even when very low (< 1.0 mmol/l) levels of LDL-cholesterol (LDL-C) have been achieved [6–10]. Notably, this residual ASCVD risk has been reported to be especially high in patients with type 2 diabetes [11, 12]. Emerging evidence has highlighted the contribution that elevated levels of triglyceride (TG)-rich lipoproteins (TRLs) appear to make to this ongoing high ASCVD risk [6, 7, 10, 11, 13–15]. TRLs, which include lipoproteins of both liver (apolipoprotein [Apo]B-100-containing VLDL) and intestinal (ApoB-48-containing chylomicrons and VLDL) origin, are believed to have a causal role in atherosclerosis. The most compelling evidence for this comes from studies showing that genetic variants that perturb their plasma levels are linked to altered risk of ASCVD. Thus, in order to develop effective prevention strategies beyond LDL-C lowering that address the residual risk linked to raised TG, there is an urgent need to better understand the structure and metabolism of TRLs in order to identify potential new targets for intervention [9, 16].

Elevated plasma concentrations of chylomicrons and VLDL, particularly their partial lipolysis products—remnants and intermediate-density lipoproteins (IDLs)—have been associated with increased ASCVD risk in epidemiological studies and clinical trials [14, 17–19]. At present, however, it is unclear as to whether lowering the levels of these particles will lead to a further reduction in ASCVD risk. So far, the evidence from TG-lowering outcome trials has been inconsistent. The promise seen in early trials of TG lowering [20, 21] has not been replicated in recent studies, especially when individuals were on background statin therapy [22, 23]. In the most recent study, the Pemafibrate to Reduce Cardiovascular Outcomes by Reducing Triglycerides in Patients with Diabetes (PROMINENT) trial, which used pemafibrate in individuals with type 2 diabetes with raised TG levels, there was a lack of clinical benefit in cardiovascular outcomes even though reductions in biomarkers (such as TRL-cholesterol [TRL-C]) were achieved that should, according to current concepts, have been associated with reduced ASCVD risk [23].

The answer as to why TG-lowering interventions like fibrates were not effective likely lies in the complexity of factors that govern TRL structure and metabolism [24]. Intravascular processing of TRL once secreted from the intestine or liver is regulated by a multiplicity of proteins that interact with enzymes and cell surface receptors. Further, as TRLs pass down the lipolytic cascade, a highly heterogeneous spectrum of remodelled lipoprotein particles of differing size, composition and probably atherogenic potential is generated [16, 19, 25–28]. Better understanding of the causes and consequences of dysregulation of TRL metabolism in the fasted and postprandial states, especially in individuals with type 2 diabetes, is essential in order to uncover the basis of residual risk [17, 29, 30] and how best to intervene [9, 17, 26, 31–35]. To this end, the present study investigates in individuals with type 2 diabetes on statin therapy the entire spectrum of ApoB-48- and ApoB-100-containing lipoproteins using an integrated non-steady-state model [36, 37]. Our central hypothesis was that dysregulation of the metabolism of intestinally derived lipoproteins is central to the abnormality in TG metabolism seen in type 2 diabetes, and the associated high and persistent ASCVD risk.

## Methods

### Study participants

To investigate the kinetics of ApoB-48- and ApoB-100-containing lipoproteins, we performed a secondary analysis of 11 overweight/obese individuals with type 2 diabetes treated with lifestyle counselling and on a stable dose of metformin who were from an earlier clinical study (ClinicalTrials.gov registration no. NCT02948777; Helsinki University research portal no. 5787) [38], and compared these with 11 control participants frequency-matched for age, BMI and sex (Table 1). The control participants were newly recruited through newspaper advertisement. Ten out of eleven control participants were on statins already at screening. Sex and race/ethnicity data were obtained from the participants’ national identification number. In the individuals with diabetes, the dose of metformin was constant for at least 4 weeks before performing kinetic studies, and was either 1000 mg (*n*=2 participants), 1500 mg (*n*=6 participants), 2000 mg (*n*=2 participants) or 3000 mg (*n*=1 participant) per day. This range in metformin dose was permitted in the protocol to achieve and maintain individualised good glycaemic control as no other glucose-lowering medications were allowed. All participants were receiving in-study statin treatment at the following doses: atorvastatin, 10–40 mg/day (*n*=18 participants), simvastatin, 20 mg/day (*n*=2 participants) and rosuvastatin 10 mg/day (*n*=2 participants). On these statin regimens LDL target levels were achieved in both the control participants and individuals with type 2 diabetes. Any other medications taken by the study participants were not considered to affect lipoproteins.

**Table 1:** Characteristics of control individuals and individuals with type 2 diabetes on statin treatment at baseline

Characteristics	Control (*n*=11)	Type 2 diabetes (*n*=11)	*p* value^a^
Sex (men/women)	4/7	5/6	
Age (years)	66.7±5.9	66.1±5.9	0.82
Weight (kg)	85.2±12.5	78.8±10.7	0.22
BMI (kg/m^2^)	29.5±2.4	28.6±3.1	0.24
Waist (cm)	102±10	102±8	0.77
Liver fat (%)	3.46±3.75	4.22±3.26	0.32
VAT (cm^3^)	1940±760	2200±680	0.44
SAT (cm^3^)	4380±1400	3840±1900	0.30
Systolic BP (mmHg)	135±17	138±17	0.58
Diastolic BP (mmHg)	80.0±8.7	82.2±6.1	0.41
Heart rate (bpm)	65.9±8.6	67.2±10.1	0.90
Glycaemic indices			
Plasma glucose (mmol/l)	5.24 ± 0.30	6.22±0.35	< 0.0001
HbA_1c_ (mmol/mol)	37.3±2.0	40.6±4.0	0.04
HbA_1c_ (%)	5.56±0.19	5.86±0.36	0.04
Insulin (pmol/l)	54.7±25.3	53.8±17.7	0.90
HOMA2^b^			
HOMA2-%B	56±46	55±13	0.17
HOMA2-%S	150±120	126±61	0.75
HOMA2-IR	1.01±0.55	0.92±0.30	0.75
Plasma lipid profile			
Cholesterol (mmol/l)	4.02±0.62	3.77±0.42	0.56
LDL*-*C (mmol/l)	1.71±0.28	1.62±0.33	0.33
HDL*-*C (mmol/l)	1.70±0.49	1.44±0.41	0.24
TGs (mmol/l)	1.09±0.40	1.46±0.45	0.06
ApoC-III (g/l)	0.106±0.023	0.116±0.043	0.95
ApoB (g/l)	0.655±0.078	0.714±0.100	0.19
ApoB*-*48 (g/l)	0.069±0.037	0.059±0.036	0.70
ApoE (g/l)	0.030±0.011	0.031±0.008	0.97
ApoA-I (g/l)	1.59±0.25	1.42±0.27	0.09
Lp(a) (nmol/l)	71.0±72.3	55.4±54.6	0.95
NEFA (mmol/l)	0.57±0.22	0.64±0.18	0.61
B-OHB (μmol/l)	100.2±58.2	67.4±22.6	0.19
LPL mass (ng/ml)	413±150	443±120	0.37
ANGPTL3 (ng/ml)	84±19	66±18	0.03

Data are presented as mean±SD

^a^*p* values were calculated using the Mann–Whitney U test

^b^HOMA2 estimates steady-state beta cell function (HOMA2-%B), insulin sensitivity (HOMA2-%S) and insulin resistance (HOMA-IR)

B-OHB, β-hydroxybutyrate; HDL-C, HDL-cholesterol; Lp(a), lipoprotein a; SAT, subcutaneous adipose tissue; VAT, visceral adipose tissue

### Inclusion and exclusion criteria

Lipid level-associated inclusion criteria were applied at the screening visit for both groups and consisted of plasma TG 1.0–4.5 mmol/l and LDL-C 1.8–4.0 mmol/l on statin. The age and BMI ranges were 56–75 years and 25.1–36.2 kg/m^2^, respectively. Exclusion criteria included type 1 diabetes, HbA_1c_ > 75 mmol/mol (9.0%), diabetes medication beyond diet and metformin, ApoE2/2 genotype, fasting TG > 4.5 mmol/l, total cholesterol > 7.0 mmol/l, abnormal liver or thyroid tests, untreated or inadequately treated hypertension, history/diagnosis of diabetic nephropathy/retinopathy, estimated glomerular filtration rate < 60 ml/min per 1.73 m^2^, use of lipid-lowering drugs other than statins within 3 months of enrolment, history of ASCVD events or revascularisation procedures within the previous 6 months, congestive heart failure New York Heart Association (NYHA) class III/IV, current use of antithrombotic or anticoagulant therapy, known bleeding tendency and history of cancer within the last 5 years. The study protocol was approved by the ethics committee of Helsinki University Hospital and the National Agency of Medicines, Helsinki, Finland (Eruct 2016-00176-30). The trial was undertaken in accordance with the Declaration of Helsinki and the European Medicines Agency Note for Guidance on Good Clinical Practice. Study participants signed informed consent before study-related procedures were initiated. The insulin sensitivity (HOMA2-%S) and insulin secretion (HOMA2-%B) indexes were calculated using HOMA2 (the updated computer model for pairs of fasting glucose and insulin) [39].

### Metabolic study protocol

The kinetic studies were performed as previously reported [36–38]. Briefly, all participants were admitted to the clinical research unit of the Helsinki University Hospital on the test-meal day (day 0) after a 12 h overnight fast. Stable isotope-labelled tracers were administered to follow the kinetics of ApoB-48, ApoB-100, ApoC-III, ApoE and TGs. Participants received a bolus injection at 08:00 hours (0 h timepoint) of ^2^H-labelled leucine (5,5,5-D_3_; Eurisotop, Saint-Aubin, France; [D_3_]leucine) at a dose of 7 mg per kg body weight and a fixed dose of 500 mg of ^2^H-labelled glycerol (1,1,2,3,3-D_5_; Eurisotop; [D_5_]glycerol). Two hours after tracer administration, participants consumed a standard fat-rich meal containing 3879 kJ (927 kcal; 68 g fat, 63 g carbohydrate and 40 g protein) and comprising bread, cheese, ham, boiled eggs, fresh red pepper, a cocoa emulsion containing 40 g of olive oil (Carapelli Firence, Florence, Italy), orange juice, and tea or coffee. The meal was consumed within 15 min. During the next 8 h only water was allowed ad libitum, and the participants remained physically inactive. The participants were instructed to avoid alcohol and strenuous exercise for the 72 h before the study visit.

### Lipoprotein isolation and biochemical analyses

Lipoprotein fractions (chylomicrons, Svedberg flotation rate [*S*_f_] > 400; large VLDL_1_ particles, *S*_f_ 60–400; and smaller VLDL_2_ particles, *S*_f_ 20–60) were isolated from blood samples using density gradient ultracentrifugation [40]. IDL and LDL fractions were isolated by sequential centrifugation [39]. TG and cholesterol concentrations were analysed using the Konelab 60i analyser (Thermo Fisher Scientific, Finland). ApoB-48 levels in total plasma were measured by ELISA (Shibayagi, Shibukawa, Japan) and in lipoprotein fractions ApoB-48 was measured using mass spectrometry [36, 37]. Concentrations of plasma glucose were measured using the hexokinase method and insulin using sandwich immunoassays (Roche Diagnostics, Germany). Plasma levels of ApoC-III were measured immunoturbometrically (Kamiya Biomedical Company, Seattle, WA, USA). ELISAs were used to measure serum ApoE (STA-367, Cell Biolabs, San Diego, CA, USA) and angiopoietin-like protein 3 (ANGPTL3; RD191092200R, BioBendor, Brno, Czech Republic). β-Hydroxybutyrate concentrations were measured by an enzymatic method with the β-Hydroxybutyrate 21 FS kit (DiaSys Diagnostic Systems, Holzheim, Germany) on a Konelab 60i analyser (Thermo Fisher Scientific). Plasma NEFA were analysed with an automated enzymatic colorimetric method (Wako Chemicals, Neuss, Germany). Lipoprotein lipase (LPL) mass in plasma was measured by ELISA assay (ImmBioMed, Germany).

### Tracer enrichment in apolipoproteins and TG

[D_3_]leucine tracer enrichment in plasma was measured during the first 24 h. [D_3_]leucine enrichment in ApoB-48 in the chylomicrons, VLDL_1_ and VLDL_2_ fractions, [D_3_]leucine enrichment in ApoB-100 in VLDL_1_, VLDL_2_, IDL and LDL, and [D_5_]glycerol enrichment in VLDL_1_ and VLDL_2_ TGs were measured using mass spectrometry, as described previously [37, 41]. Enrichment of [D_3_]leucine in ApoC-III and ApoE was also determined [38, 42].

### Multicompartmental modelling and parameter estimation

Modelling of postprandial kinetics of ApoB-48- and ApoB-100-containing lipoproteins was performed using the non-steady-state multicompartmental model developed in earlier reports [36, 37]. The model was adapted to include IDL and LDL ApoB-100 kinetics as described [38]. All participants were modelled individually using Simulation Analysis and Modeling version 2.0 (SAAMII) [43]. ApoC-III and ApoE kinetics were analysed as previously described [38, 42].

### Statistics

Statistical analyses were performed using R version 3.6.3 (http://www.r-project.org). The *p* values were calculated using the Mann–Whitney *U* test. Repeated measures ANOVA was performed using the packages *lme4* and *lmerTest.* Repeated measures correlation analyses were performed using the package *rmcorr*.

## Results

The participants of this study consisted of 11 control individuals (*n*=4 men and *n*=7 women) on statin therapy (20 mg atorvastatin or equivalent), and 11 individuals (*n*=5 men and *n*=6 women) with type 2 diabetes who were on a similar statin regimen. Characteristics of the metabolic status of participants are given in Table 1. There was no evidence of a difference between the groups in weight, BMI, or degree of visceral or subcutaneous adiposity. Mean liver fat was 3.46±3.75% in control participants and 4.22±3.26% in the diabetic group. Glycaemic indicators exhibited the expected differences, with individuals with type 2 diabetes having higher blood glucose and HbA_1c_. However, the individuals with type 2 diabetes were in good glycaemic control and HOMA indices were similar in the two groups. There was also no evidence of a difference in fasting lipoprotein profiles between the two groups of participants. Notably, mean plasma TG was not statistically different and was within the normal range in both groups (although the individuals with type 2 diabetes showed a higher mean value as might be expected), as were LDL-C (low in both groups due to statin therapy), plasma ApoB and plasma ApoB-48 concentrations. LPL mass and ApoC-III were the same but ANGPTL3 was lower in the group with type 2 diabetes compared with control participants. Overall, the two groups had background characteristics that allowed us to address the question as to whether postprandial lipid responses were perturbed in well-controlled, statin-treated individuals with type 2 diabetes, and how this might contribute to lipoprotein-associated residual risk.

### Response of statin-treated control individuals and individuals with type 2 diabetes to a standard fat-rich meal

**Fig. 1:**
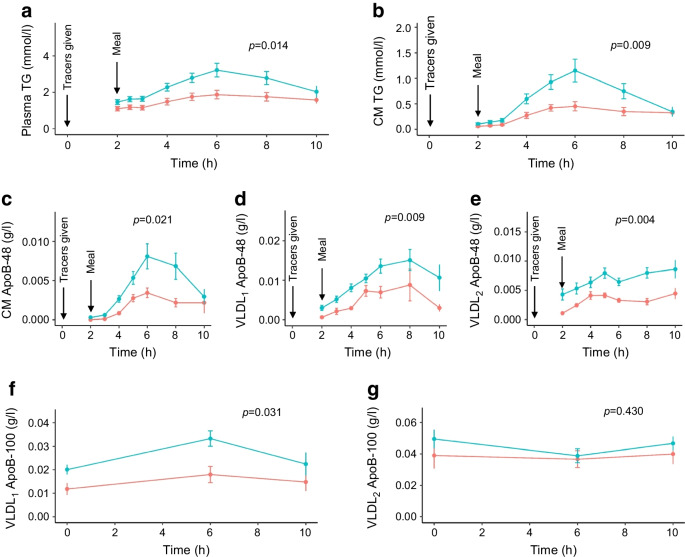
Response to standard fat-rich meal in control individuals and individuals with type 2 diabetes on statins. The metabolic protocol began at the 0 h timepoint with the injection of [D_3_]leucine and [D_5_]glycerol tracers. At the 2 h timepoint, participants consumed a standard fat-rich meal within 15 min. Blood samples were taken immediately before the meal and at frequent intervals thereafter for 8 h (ending at the 10 h timepoint) to quantify the postprandial response in (**a**) plasma TG, (**b**) chylomicron (CM) TG. In addition, the ApoB-48 concentration in the (**c**) CM, and (**d**) VLDL_1_ and (**e**) VLDL_2_ density intervals was also assessed at the same timepoints. The content of ApoB-100 in (**f**) VLDL_1_ and (**g**) VLDL_2_ was determined at 0 h, 6 h and 10 h. Responses in the control individuals and individuals with type 2 diabetes were compared using repeated measures ANOVA and by determining the AUC (see AUC data in Table 2)

All participants consumed a standard fat-rich meal at the 2 h timepoint in the metabolic protocol, and the development of postprandial lipaemia was followed over the subsequent 8 h. Postprandial responses are shown in Fig. 1. Despite the similarity in fasting plasma TG levels (Table 1), the rise in plasma and chylomicron TG was significantly greater in the individuals with type 2 diabetes than in control individuals (Fig. 1a,b). Similarly, the increment in ApoB-48 in the chylomicron, VLDL_1_ and VLDL_2_ fractions was significantly larger in the individuals with type 2 diabetes compared with control individuals (Fig. 1c–e). This inter-group difference in response for intestinally derived TG-rich lipoproteins contrasts with the observation that fasting levels and changes in VLDL_1_ and VLDL_2_ ApoB-100 were similar in control individuals and individuals with type 2 diabetes (Fig. 1f,g).

The calculated AUC (mg/l×h or mmol/l×h) responses reveal that the individuals with type 2 diabetes had significantly (approximately twofold) greater responses to the standard meal compared with control participants (Table 2). This was the case for TG in plasma and chylomicrons, and for ApoB-48 in the chylomicron, VLDL_1_ and VLDL_2_ fractions.

**Table 2:** Postprandial responses (AUC and incremental AUC) to a standard fat-rich meal in control individuals and individuals with type 2 diabetes on statin treatment

Variable	Control (*n*=11)	Type 2 diabetes (*n*=11)	*p* value^a^
AUC			
Plasma TG (mmol/l×h)	12.9±4.9	19.9±7.0	0.013
CM TG (mmol/l×h)	2.5±1.4	5.3±2.9	0.008
CM ApoB*-*48 (mg/l×h)	14.0±9.4	37.4±24.6	0.021
VLDL_1_ ApoB*-*48 (mg/l×h)	47.7±31.4	86.6±43.5	0.038
VLDL_2_ ApoB*-*48 (mg/l×h)	25.4±11.6	56.0±22.7	0.005
VLDL_1_ ApoB*-*100 (mg/l×h)	154±103	271±101	0.014
VLDL_2_ ApoB*-*100 (mg/l×h)	380±207	436±15	0.484
LDL ApoB*-*100 (mg/l×h)	3260±805	3721±716	0.172
VLDL_1_ TG (mmol/l×h)	4.2±2.5	7.3±3.0	0.017
VLDL_2_ TG (mmol/l×h)	2.2±0.7	2.7±0.6	0.124
iAUC			
Plasma TG (mmol/l×h)	4.1±2.5	8.2±4.0	0.009
CM TG (mmol/l×h)	2.0±1.1	4.5±2.4	0.006
CM ApoB*-*48 (mg/l×h)	12.5±10.6	35.1±23.7	0.009
VLDL_1_ ApoB*-*48 (mg/l×h)	35.3±33.7	62.4±33.9	0.074
VLDL_2_ ApoB*-*48 (mg/l×h)	11.5±11.0	21.7±8.6	0.025
VLDL_1_ ApoB*-*100 (mg/l×h)	36.8±35.5	71.0±50.9	0.083
VLDL_2_ ApoB*-*100 (mg/l×h)	−10.3±99.2	−58.8±86.1	0.236
VLDL_1_ TG (mmol/l×h)	1.3±1.2	2.6±1.8	0.052
VLDL_2_ TG (mmol/l×h)	0.3±0.2	0.2±0.4	0.458

^a^*p* values were calculated using the Mann–Whitney *U* test

CM, chylomicrons; iAUC, incremental AUC

### ApoB-48 and ApoB-100 kinetics in statin-treated control individuals and individuals with type 2 diabetes

**Fig. 2:**
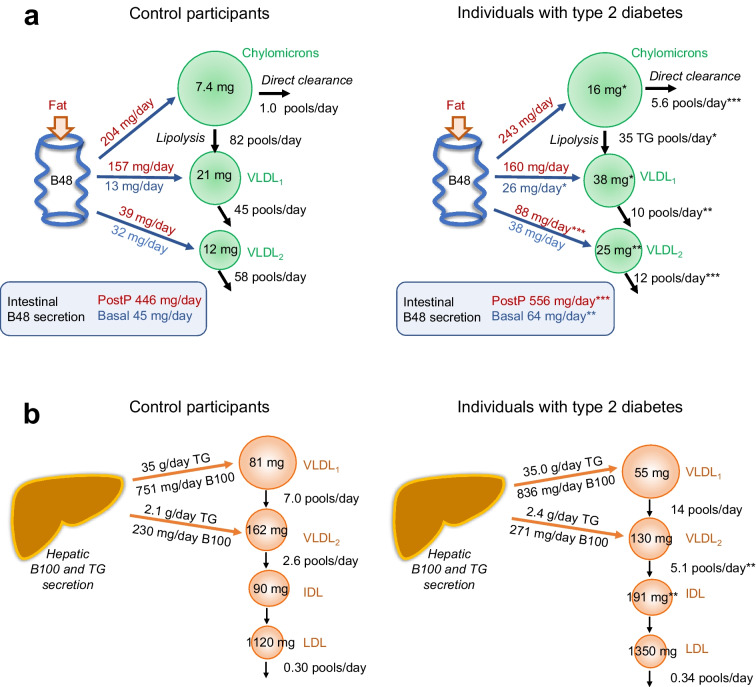
Summary of ApoB-48 and ApoB-100 kinetics in control individuals and individuals with type 2 diabetes on statins. Key kinetic rate constants are shown for the metabolism of (**a**) ApoB-48 and (**b**) ApoB-100. Production rates are given in mg/day and conversion (fractional transfer rates) or clearance rates in pools/day. The ApoB-48 pool size for chylomicrons, VLDL_1_ and VLDL_2_ (**a**) was determined as the time-averaged concentration of ApoB-48 (summed concentrations divided by number of timepoints) across the postprandial (PostP) period (2–10 h, as shown in Fig. 1c–e). Total ApoB-48 production rates into all density intervals are given in blue boxes in schematic (**a**), for both the basal state (fasting) and for the PostP period. Basal production, blue text; PostP production, red text. In (**b**), pool sizes (in mg) were determined for VLDL_1_, VLDL_2_, IDL and LDL from the average measured plasma concentration of these ApoB-100 lipoproteins. B48, ApoB-48; B100, ApoB-100. *p < 0.05, **p < 0.01 and ***p < 0.001 vs control, calculated using the Mann–Whitney *U* test

Kinetic parameters describing the metabolism of ApoB-48 and ApoB-100 in the two groups were derived by applying a non-steady-state compartmental model to the data shown in electronic supplementary material (ESM) Fig. 1 and ESM Table 1, which included baseline concentrations and postprandial responses in TG, ApoB-48 and ApoB-100 in the various lipoprotein fractions, and the tracer enrichment data for ApoB-48, ApoB-100 and TG. Production, interconversion and clearance rates for the individual fractions (chylomicrons, VLDL_1_, VLDL_2_, IDL and LDL) are given in ESM Table 1 and key parameters are summarised in Fig. 2.

The most notable differences in ApoB metabolism in the two groups lay clearly in the kinetics of intestinally derived ApoB-48-containing lipoproteins. Basal production of ApoB-48 (that is, in the fasting state prior to meal consumption) was 42% higher, and the total ApoB-48 secreted during the postprandial response was 25% higher (556 ± 57 vs 446 ± 57 mg/day; *p *< 0.001), in individuals with type 2 diabetes vs control individuals (Fig. 2). Within the VLDL density interval, VLDL_1_ ApoB-48 production rate was higher in the basal state, and VLDL_2_ ApoB-48 production higher post-prandially, in the individuals with type 2 diabetes. The fractional rate of transfer of chylomicron particles into ApoB-48-containing VLDL_1_ was around 40% lower in individuals with type 2 diabetes compared with control individuals (35 ± 25 vs 82 ± 58 pools/day; *p*=0.034), and the fractional clearance rates of ApoB-48-containing VLDL_1_ and VLDL_2_ were also markedly lower (Fig. 2, ESM Table 1). Conversely, the fractional rate of direct clearance of chylomicron particles was greater, at 5.6±2.2 pools/day in individuals with type 2 diabetes vs 1.0±1.8 pool/day in control participants (*p *< 0.001). As a result of these differences in ApoB-48 kinetics, the mean pool size for ApoB-48 in chylomicrons, VLDL_1_ and VLDL_2_ was significantly higher in the group with type 2 diabetes compared with control values (ESM Table 2). Further, when ApoB pool sizes were expressed on a per-particle basis (that is, in nmol/l allowing for the difference in molecular weight of ApoB-48 and ApoB-100), it was clear that across the 8 h of the postprandial period ApoB-48-containing lipoproteins in the VLDL density interval were a much higher proportion of the total VLDL present in the individuals with type 2 diabetes (at 44%) than in the control individuals (at 25%) (ESM Table 2). These ApoB-48 VLDL particles in the individuals with type 2 diabetes had a prolonged residence time in the circulation of the order of 2.0–2.4 h compared with about 0.5 h in control participants (residence time is 1/fractional catabolic rate [FCR] for VLDL_1_ ApoB-48 and VLDL_2_ ApoB-48 in ESM Table 1).

In contrast, the rates of production of ApoB-100 and TG in hepatic-derived ApoB-100-containing VLDL_1_ and VLDL_2_ showed no evidence of a difference in the two groups (Fig. 2b). The only significant differences were seen for IDL, where the pool size was about twofold higher in individuals with type 2 diabetes (ESM Table 2). This was attributable to a greater rate of conversion of VLDL_2_ to IDL (ESM Table 1). Notably, the rates of LDL ApoB-100 production and clearance were similar in the two groups (ESM Table 1), as was the LDL pool size (ESM Table 2).

### Metabolism of ApoC-III and ApoE

The production and clearance rates for ApoC-III and ApoE showed no evidence of a difference between the individuals with type 2 diabetes and control individuals (ESM Table 3).

## Discussion

The aim of this study was to explore the nature of lipoprotein-associated residual risk in statin-treated individuals with type 2 diabetes. We found clear evidence that intestinal lipoprotein metabolism displayed distinct abnormalities in individuals with type 2 diabetes compared with control individuals on similar statin treatment regimens. Specifically, participants with type 2 diabetes exhibited a greater response to a standard fat-rich meal, with higher levels of chylomicron TG and ApoB-48 during the postprandial phase. The concentration of ApoB-48-containing particles in the VLDL_1_ and VLDL_2_ fractions was also about twofold higher in individuals with type 2 diabetes during fat absorption. The kinetic basis of these differences between the groups was found to be a combination of overproduction of ApoB-48-containing lipoproteins secreted into the chylomicron and VLDL density ranges by the intestine, a lower lipolysis rate for chylomicrons (as reflected in the ApoB-48 fractional transfer rate to VLDL_1_) and lower clearance rates for ApoB-48-containing VLDL in the individuals with type 2 diabetes. We noted, however, that direct clearance of chylomicrons in individuals with type 2 diabetes occurred at a higher rate than in control individuals. In contrast, no significant differences were seen in the kinetics of liver-derived ApoB-100-containing VLDL between the groups. These findings indicate that a substantial element of the residual risk on statin therapy may be attributed to perturbations in the metabolism of intestinally derived lipoproteins, abnormalities that are not reflected in the fasting lipid profile. Since clinical trials reveal a considerable residual risk in individuals with type 2 diabetes on statin or even combination lipid-lowering therapy [11], the results of the present study highlight the importance of targeting intestinal lipoprotein processing as a means of addressing this unmet need.

Overproduction of ApoB-48-containing lipoproteins by the intestine in individuals with type 2 diabetes has been reported previously [44–46]. This was seen in both the response to a fat-rich meal and in studies of ApoB-48 kinetics conducted in a quasi-steady state induced by feeding micro-meals across the day [45]. These earlier investigations, however, did not address directly the critical question explored here. That is, are there in individuals with type 2 diabetes who are in good glycaemic control and on statin treatment persisting abnormalities in ApoB-48 kinetics that help explain the residual risk of ASCVD, especially in individuals with normal fasting plasma TG levels. Elevations in plasma TG and TRL are a well-known feature of type 2 diabetes, particularly in newly diagnosed or poorly controlled individuals, due to a combination of elevated VLDL_1_ secretion by the liver and delayed lipolysis [47]. Insulin is recognised as a major regulator of hepatic VLDL assembly and secretion and lipolysis, and when glycaemic control is optimised plasma TG levels fall due to correction of these metabolic abnormalities. The fact that we observed only modest, if any, between-group differences in plasma TG, in VLDL_1_ and VLDL_2_ ApoB-100 pool sizes, or in VLDL_1_ and VLDL_2_ ApoB-100 and TG kinetics is likely a reflection of the good glycaemic control achieved in the individuals with type 2 diabetes. Insulin appears also to be an important regulator of ApoB-48 synthesis and the assembly and secretion of TG-rich lipoproteins in the intestine [48]. Acute administration of the hormone leads to a decrease in ApoB-48 synthesis, and the rate of ApoB-48 production is strongly related to the degree of insulin resistance [44, 48, 49]. It is noteworthy, therefore, in the present study that a higher ApoB-48 production persisted in the group of participants with type 2 diabetes even though VLDL ApoB-100 synthesis rates were virtually the same as in control participants. It may be that the sensitivity of the ApoB-48 lipoprotein production pathway in the intestine to insulin control is less than that of the ApoB-100 VLDL assembly and secretion pathway in the liver, or that other major gut hormones such as incretins influence and modify the action of insulin [48, 50].

A number of drugs used to regulate blood glucose levels in individuals with type 2 diabetes may influence postprandial lipid metabolism [44] and it is for this reason that the individuals we studied with type 2 diabetes were all on metformin as the sole agent used for glycaemic control. Metformin has been reported in some but not all studies to reduce postprandial levels of TGs, chylomicrons and chylomicron remnants [44, 51–53]. The potential mechanisms of action have been reported to involve a direct effect of metformin on the expression of genes involved in intestinal lipid metabolism [54], delayed gastric emptying [55] and increased glucagon-like peptide 1 (GLP-1) secretion [56].

Statin treatment has profound effects on ApoB metabolism, increasing the activity of LDL receptors and enhancing clearance of ApoB-100-containing lipoprotein particles from the circulation. Metabolic studies have shown that statins increase the fractional clearance rates of VLDL_1_, VLDL_2_, IDL and LDL in hypercholesterolaemic and other individuals, including individuals with type 2 diabetes [57, 58]. Both groups in the present study were on a similar statin regimen and it is to be expected that kinetic variables related to lipoprotein removal rates would reflect this fact. No significant differences were seen in the fractional clearance rates for VLDL_1_, VLDL_2_, IDL and LDL ApoB-100 particles. The only difference recorded was in the pool size of IDL ApoB-100, which was higher in the individuals with type 2 diabetes due to increased conversion from VLDL_2_ and may indicate the accumulation of remnants of VLDL lipolysis in this density range [17]. Statins do affect the postprandial response to a fat-rich meal. The AUC has been shown to be lower on statin therapy, and this has been attributed to increased clearance of chylomicrons and their remnants as a result possibly of stimulated receptor-mediated catabolism [59, 60]. Since LDL-C and plasma TG reductions on statins in individuals with type 2 diabetes are generally quantitatively similar to those in control individuals [61, 62], it is likely that the statin regimen was not responsible for the differences in postprandial response seen between the two groups in our study. Rather, the abnormalities we observed in individuals with type 2 diabetes are likely due to an inherently perturbed metabolism of intestinally derived lipoproteins that persists even on a background of effective statin treatment.

The extent to which the abnormalities in intestinal lipoprotein metabolism explain residual risk in individuals with type 2 diabetes on statin therapy is unknown at present. Chylomicron remnants are believed to be atherogenic based on epidemiological and animal model studies but, so far, we lack a quantitative estimate as to the contribution of plasma levels of ApoB-48-containing chylomicron remnants and VLDL particles to ASCVD risk. What is clear from the present investigation is that recording a fasting plasma TG level in the ‘normal’ range does not mean that TG metabolism can be considered unremarkable and unlikely to contribute to atherosclerosis development. To what degree this cryptic metabolic abnormality helps explain the results of recent clinical trials is also an unknown. In the Reduction of Cardiovascular Events with Icosapent Ethyl–Intervention Trial (REDUCE-IT), the reduction in ASCVD risk was unrelated to the fasting plasma TG level, and other mechanisms of benefit have been suggested [63]. However, it is evident from kinetic studies that high-dose eicosapentaenoic acid (EPA) can substantially reduce the production of ApoB-48-containing lipoproteins from the intestine, and this action warrants further investigation in the light of our findings [64]. Other drugs have been shown to alter ApoB-48 synthesis in the intestine. Recent studies, one of which used the same protocol as employed here [49], have shown that GLP-1 receptor agonists such as liraglutide at pharmacological doses inhibit ApoB-48-containing chylomicron production [48, 49]. We found also that the incretin reduced direct clearance of chylomicron particles. How much this normalisation of chylomicron metabolism contributes to the finding that GLP-1 receptor agonists reduce incidence of CVD in outcome trials deserves further investigation.

The main weakness of the present study is the limited number of participants which reflects the complexity of the metabolic protocol and the demands placed on volunteers. All participants with type 2 diabetes were on metformin and statin therapy, and the results should be evaluated in light of this therapeutic setting. The findings may not be generalisable to individuals with type 2 diabetes on other glycaemic and lipid-lowering regimens. That said, this treatment combination is regarded as a ‘cornerstone of diabetes therapy’ for the large, and rapidly growing, group of patients with type 2 diabetes [65–67], and our results provide insight into persistent issues relating to ASCVD risk for a substantial proportion of real-world patients diagnosed with type 2 diabetes [68]. We and others in previous publications have extensively investigated the abnormalities in lipoprotein metabolism in individuals with more pronounced type 2 diabetes and have shown increased ApoB-48 production by the intestine and demonstrated that GLP-1 receptor agonists reduce the intestinal ApoB-48 overproduction [44, 49, 69].

In summary, evaluation of the kinetics of ApoB-48-containing lipoproteins revealed that characteristic abnormalities in the metabolism of intestinally derived lipoprotein in individuals with type 2 diabetes were present even when on statin therapy. Compared with control individuals, individuals with type 2 diabetes: (1) exhibited higher production rates for ApoB-48-containing particles secreted in the form of chylomicrons and VLDL; (2) had delayed lipolysis of chylomicrons; and (3) accumulated ApoB-48 VLDL with a prolonged residence time in the circulation. These perturbations in the postprandial response resulted in twofold higher postprandial levels of chylomicrons, ApoB-48 VLDL and their remnants, with potential consequences for atherosclerosis. Importantly, the fasting lipid profile showed no evidence of a difference between the two groups we examined, although there was a non-significant trend to higher mean plasma TG in the individuals with type 2 diabetes; even then, both levels were well within the normal range. The implication of our study is that much greater investigation of postprandial lipoprotein structure, metabolism and particle atherogenicity is required to better understand the basis of residual risk in statin-treated individuals and, possibly, the results of cardiovascular outcome trials. New targets for intervention will undoubtedly arise as this cryptic risk factor is made more overt.

### Supplementary Information

Below is the link to the electronic supplementary material.

Supplementary file1 (PDF 871 KB)

## Data Availability

Data are available on a population basis but not on an individual level. This requirement is a condition of the ethical permission for the study.

## Abbreviations

ANGPTL3: Angiopoietin-like protein 3
Apo: Apolipoprotein
ASCVD: Atherosclerotic CVD
GLP-1: Glucagon-like peptide 1
IDL: Intermediate-density lipoprotein
LDL-C: LDL-cholesterol
LPL: Lipoprotein lipase
S_f_: Svedberg flotation rate
TG: Triglyceride
TRL: Triglyceride-rich lipoprotein
