# Supplementary material for: Contribution of intestinal triglyceride-rich lipoproteins to residual atherosclerotic cardiovascular disease risk in individuals with type 2 diabetes on statin therapy
Source: Diabetologia. 2023 Sep 29;66(12):2307–19. doi: 10.1007/s00125-023-06008-0 (PMC10627993; doi:10.1007/s00125-023-06008-0)
Supplement: Supplementary file 1 — Supplementary file1 (PDF 871 KB) [file 125_2023_6008_MOESM1_ESM.pdf]

SUPPLEMENTARY FILE

Contribution of intestinal triglyceride-rich lipoproteins to residual atherosclerotic cardiovascular disease risk in individuals with type 2 diabetes on statin therapy

Marja-Riitta Taskinen, Niina Matikainen, Elias Björnson, Sanni Söderlund, Jussi Inkeri, Antti Hakkarainen, Helka Parviainen, Carina Sihlbom, Annika Thorsell, Linda Andersson, Martin Adiels, Chris J Packard and Jan Borén

ESM Table 1. ApoB-48 and ApoB-100 kinetic rate constants for control subjects and individuals with type 2 diabetes.

	Control participants Mean ± SD, (N=11)	Type 2 diabetes Mean ± SD, (N=11)	P-value*
**ApoB-48 production rates**			
ApoB-48 total prod (mg/day)	446 ± 57	556 ± 57	< 0.001
ApoB-48 CM postprandial prod (mg/day)	204 ± 47	243 ± 51	0.057
ApoB-48 VLDL1 postprandial prod (mg/day)	157 ± 47	160 ± 66	0.90
ApoB-48 VLDL2 postprandial prod (mg/day)	39 ± 7.2	88 ± 32	< 0.001
ApoB-48 VLDL postprandial prod (mg/day)	196 ± 53	248 ± 67	0.076
ApoB-48 basal prod (mg/day)	45 ± 11	64 ± 7.6	0.001
ApoB-48 basal VLDL1 prod (mg/day)	13.4 ± 4.8	26.2 ± 4.8	< 0.0001
ApoB-48 basal VLDL2 prod (mg/day)	31.9 ± 7.1	37.9 ± 5.9	0.17
ApoB-48 basal TG prod (g/day)	0.63 ± 0.24	2.49 ± 0.34	< 0.0001
**ApoB-100 production rates**			
ApoB-100 VLDL1 prod (mg/day)	751 ± 76	836 ± 230	0.057
ApoB-100 VLDL2 prod (mg/day)	617 ± 210	805 ± 330	0.33
ApoB-100 VLDL2 direct prod (mg/day)	230 ± 94	271 ± 84	0.47
ApoB-100 VLDL total prod (mg/day)	981 ± 95	1110 ± 300	0.15
ApoB-100 LDL prod (mg/day)	430 ± 140	438 ± 190	0.90
ApoB-100 TG VLDL1 prod (g/day)	35 ± 10	35 ± 19	1
ApoB-100 TG VLDL2 prod (g/day)	5.8 ± 2.5	8.7 ± 4.2	0.076
ApoB-100 TG VLDL2 direct prod (g/day)	2.1 ± 0.8	2.4 ± 0.8	0.51
ApoB-100 TG VLDL total prod (g/day)	36.8 ± 9.9	37 ± 20	1
**ApoB-48 clearance rates**			
ApoB-48 Total FCR (pools/day)	72 ± 110	27 ± 10	0.016
ApoB-48 CM FCR (pools/day)	83 ± 57	40 ± 25	0.076
ApoB-48 CM FDC (pools/day)	1.0 ± 1.8	5.6 ± 2.2	< 0.001
ApoB-48 CM FTR (pools/day)	82 ± 58	35 ± 25	0.034
ApoB-48 VLDL1 FCR (pools/day)	45 ± 70	10 ± 4.6	0.0019
ApoB-48 VLDL2 FCR (pools/day)	58 ± 110	12 ± 3.9	< 0.0001
ApoB-48 TG CM FCR (pools/day)	165 ± 100	44.7 ± 27	< 0.0001
ApoB-48 TG VLDL1 FCR (pools/day)	45 ± 70	10.0 ± 4.6	0.0019
ApoB-48 TG VLDL2 FCR (pools/day)	58 ± 110	12.1 ± 3.9	< 0.0001
**ApoB-100 clearance rates**			
ApoB-100 VLDL1 FCR (pools/day)	14.1 ± 8.7	18.7 ± 8.1	0.17
ApoB-100 VLDL1 FDC (pools/day)	7.1 ± 6.5	4.5 ± 4.9	0.17
ApoB-100 VLDL1 FTR (pools/day)	7.0 ± 6.6	14 ± 11	0.22
ApoB-100 VLDL2 FCR (pools/day)	4.8 ± 3.3	6.8 ± 3.5	0.13
ApoB-100 VLDL2 FTR (pools/day)	2.6 ± 1.5	5.1 ± 2.2	< 0.01
ApoB-100 VLDL2 FDC (pools/day)	2.3 ± 2.2	1.6 ± 2.3	0.22
ApoB-100 TG VLDL1 FCR (pools/day)	28 ± 19	42 ± 27	0.15
ApoB-100 TG VLDL1 FDC (pools/day)	25 ± 19	37 ± 24	0.33
ApoB-100 TG VLDL1 FTR (pools/day)	2.5 ± 1.8	5.5 ± 4	0.10
ApoB-100 TG VLDL2 FCR (pools/day)	10.2 ± 8.3	11.2 ± 6.6	0.70
ApoB-100 IDL FCR (pools/day)	4.8 ± 3.6	3.6 ± 1.4	0.65
ApoB-100 LDL FCR (pools/day)	0.30 ± 0.2	0.34 ± 0.2	0.27

*P-values have been calculated using the Mann-Whitney U-test.

FCR – fractional clearance rate; FDC – fractional rate of direct clearance; FTR fractional transfer rate to next lipoprotein in delipidation sequence.

Direct production is the amount of ApoB secreted directly into the lipoprotein. CM- chylomicron.

Basal rates are those derived for the fasting state. Post prandial rates are derived for the post-prandial period that is from 2 to 10 hours in the protocol.

ESM Table 2. ApoB-48 and ApoB-100 pool sizes.

	Control participants Mean ± SD, (N=11)	Type 2 diabetes Mean ± SD, (N=11)	P-value*
**ApoB-48 pool sizes (mg)**			
CM B48 pool size (mg)	7.4 ± 5.1	16 ± 9.5	0.024
VLDL1 B48 pool size (mg)	21 ± 16	38 ± 17	0.035
VLDL2 B48 pool size (mg)	12 ± 5.7	25 ± 9.3	0.003
Total VLDL B48 pool size (mg)	35 ± 23	62 ± 26	0.027
**ApoB-100 pool sizes (mg)**			
VLDL1 ApoB-100 pool size (mg)	81 ± 61	55 ± 29	0.22
VLDL2 ApoB-100 pool size (mg)	162 ± 78	130 ± 48	0.27
Total VLDL ApoB-100 pool size (mg)	242± 124	185 ± 65	0.19
IDL ApoB-100 pool size (mg)	90 ± 71	191 ± 54	0.001
LDL ApoB-100 pool size (mg)	1120 ± 270	1350± 383	0.13
**ApoB-48 pool sizes (nmol)**			
CM ApoB-48 pool size (nmol)	31 ± 21	67 ± 40	0.024
VLDL1 ApoB-48 pool size (nmol)	88 ± 68	158 ± 72	0.035
VLDL2 ApoB-48 pool size (nmol)	48 ± 24	103 ± 39	0.003
Total VLDL ApoB-48 pool size (nmol)	144± 96	260 ± 108	0.027
**ApoB-100 pool sizes (nmol)**			
VLDL1 ApoB-100 pool size (nmol)	147 ± 111	100± 53	0.22
VLDL2 ApoB-100 pool size (nmol)	294 ± 142	237 ± 87	0.27
Total VLDL ApoB-100 pool size (nmol)	441 ± 225	337± 119	0.19
IDL ApoB-100 pool size (nmol)	163 ± 130	347 ± 98	0.001
LDL ApoB-100 pool size (nmol)	2045 ± 490	2450 ± 695	0.13

*P-values have been calculated using the Mann-Whitney U-test. Pool sizes were determined from repeated measurement of the ApoB-48 and ApoB-100 concentration in individual lipoprotein fractions by mass spectrometry (in mg/L) multiplied by the plasma volume (litres) as previously reported [1]. To convert mg ApoB-48 to nmol ApoB-48, a molecular weight of 240 kDa was used. To convert mg ApoB-100 to nmol ApoB-100, a molecular weight of 550 kDa was used.

ESM Table 3. Kinetic parameters of ApoE and ApoC-III in control and type 2 diabetic subjects on statin therapy.

	Control *participants* Mean ± SD, *(*N=11)	Type 2 diabetes Mean ± SD, (N=11)	P-value*
ApoE SR (mg/day)	555 ± 130	642 ± 120	0.18
ApoE FCR (pool/day)	5.2 ± 1.6	6.2 ± 1.7	0.11
ApoC-III SR (mg/day)	638 ± 330	488 ± 190	0.35
ApoC-III FCR (pools/day)	1.6 ± 0.8	1.2 ± 0.6	0.28

Kinetic constants for ApoC-III and ApoE were derived by compartmental modelling as described previously [2, 3]. SR – synthetic rate; FCR – fractional catabolic rate.

ESM Fig. 1 Metabolic data used in compartmental modelling of ApoB-48, ApoB-100 and triglyceride kinetics.

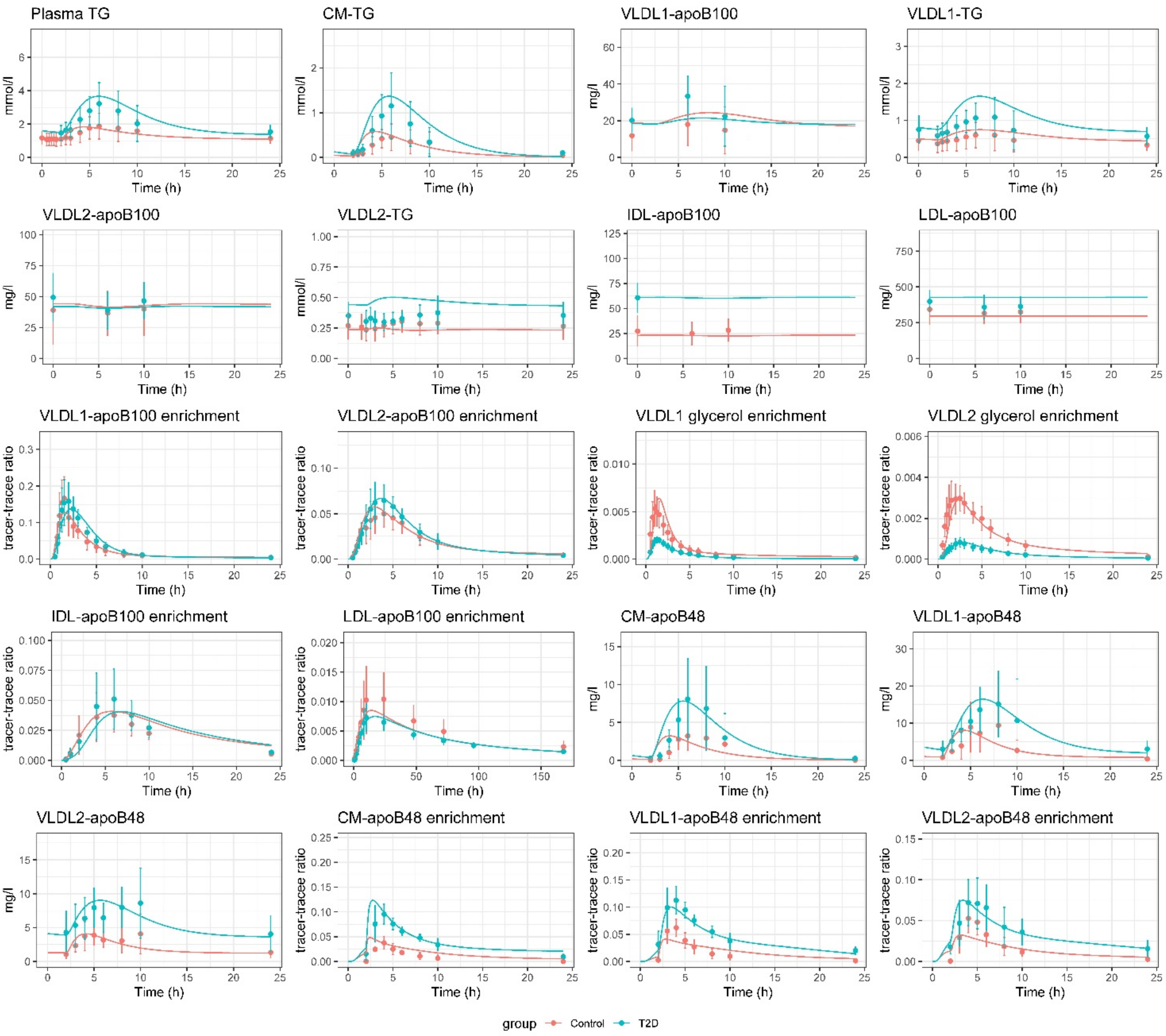

Data used to derive kinetic rate constants using the non-steady-state compartmental model described in detail in [1]. Tracers of D3-leucine and D5-glycerol were administered at the 0-hour timepoint (about 8.00 am). A fat-rich meal was consumed at the 2-hour timepoint. Blood samples were taken frequently as shown up to 10 hours and then at days 1, 2, 3, 4 and 7 to define the IDL and LDL curves.

Inputs to the model comprised triglyceride concentrations in plasma (TG), chylomicrons (CM), $VLDL_1$ and $VLDL_2$ (fasting and across the post-prandial period); ApoB-48 concentrations in chylomicrons, $VLDL_1$ and $VLDL_2$; ApoB-100 concentrations in $VLDL_1$, $VLDL_2$, IDL and LDL; D3-leucine enrichments in ApoB-48 in chylomicrons, $VLDL_1$, and $VLDL_2$; D3-leucine enrichments in ApoB-100 in.$VLDL_1$, $VLDL_2$, IDL and LDL; D5-glycerol enrichment in triglyceride in $VLDL_1$ and in $VLDL_2$. Modelling was performed using the SAAM2 programme.

The data points represent mean ± standard error values in the control group in red and individuals with type 2 diabetes in blue. Lines are the computer-generated fits to the observed data.

REFERENCES

[1] Bjornson E, Packard CJ, Adiels M, et al. (2019) Investigation of human apoB48 metabolism using a new, integrated non-steady-state model of apoB48 and apoB100 kinetics. J Intern Med 285: 562-577

[2] Taskinen MR, Bjornson E, Kahri J, et al. (2021) Effects of Evolocumab on the Postprandial Kinetics of Apo (Apolipoprotein) B100- and B48-Containing Lipoproteins in Subjects With Type 2 Diabetes. Arterioscler Thromb Vasc Biol 41: 962-975

[3] Bjornson E, Packard CJ, Adiels M, et al. (2020) Apolipoprotein B48 metabolism in chylomicrons and very low-density lipoproteins and its role in triglyceride transport in normo- and hypertriglyceridemic human subjects. J Intern Med 288: 422-438
